# Role of Dietary Amino Acids and Nutrient Sensing System in Pregnancy Associated Disorders

**DOI:** 10.3389/fphar.2020.586979

**Published:** 2020-12-22

**Authors:** Tarique Hussain, Bie Tan, Ghulam Murtaza, Elsayed Metwally, Huansheng Yang, Muhammad Saleem Kalhoro, Dildar Hussain Kalhoro, Muhammad Ismail Chughtai, Yulong Yin

**Affiliations:** ^1^ College of Animal Science and Technology, Hunan Agricultural University, Changsha, China; ^2^ Institute of Subtropical Agriculture, Chinese Academy of Sciences, Changsha, China; ^3^ Animal Sciences Division, Nuclear Institute for Agriculture and Biology College, Pakistan Institute of Engineering and Applied Sciences (NIAB-C,PIEAS), Faisalabad, Pakistan; ^4^ Department of Animal Reproduction, Faculty of Animal Husbandry and Veterinary Sciences, Sindh Agriculture University, Sindh, Pakistan; ^5^ Department of Cytology & Histology, Faculty of Veterinary Medicine, Suez Canal University, Ismailia, Egypt; ^6^ Hunan International Joint laboratory of Animal Intestinal Ecology and Health, Laboratory of Animal Nutrition and Human Health, College of Life Sciences, Hunan Normal University, Changsha, China; ^7^ Department of Animal Products Technology, Faculty of Animal Husbandry and Veterinary Sciences, Sindh Agriculture University, Sindh, Pakistan; ^8^ Department of Veterinary Microbiology, Faculty of Animal Husbandry and Veterinary Sciences, Sindh Agriculture University, Sindh, Pakistan

**Keywords:** dietary amino acids, reproductive disorders, signaling pathways, pregnancy, oxidative stress

## Abstract

Defective implantation is related to pregnancy-associated disorders such as spontaneous miscarriage, intrauterine fetal growth restriction and others. Several factors proclaimed to be involved such as physiological, nutritional, environmental and managemental that leads to cause oxidative stress. Overloading of free radicals promotes oxidative stress, and the internal body system could not combat its ability to encounter the damaging effects and subsequently leading to pregnancy-related disorders. During pregnancy, essential amino acids display important role for optimum fetal growth and other necessary functions for continuing fruitful pregnancy. In this context, dietary amino acids have received much attention regarding the nutritional concerns during pregnancy. Arginine, glutamine, tryptophan and taurine play a crucial role in fetal growth, development and survival while ornithine and proline are important players for the regulation of gene expression, protein synthesis and angiogenesis. Moreover, amino acids also stimulate the mammalian target of rapamycin (mTOR) signaling pathway which plays a central role in the synthesis of proteins in placenta, uterus and fetus. This review article explores the significances of dietary amino acids in pregnancy development, regulation of nutrient-sensing pathways such as mTOR, peroxisome proliferator-activated receptors (PPARs), insulin/insulin-like growth factor signaling pathway (IIS) and 5′ adenosine monophosphate-activated protein kinase (AMPK) which exhibit important role in reproduction and its related problems. In addition, the antioxidant function of dietary amino acids against oxidative stress triggering pregnancy disorders and their possible outcomes will also be enlightened. Dietary supplementation of amino acids during pregnancy could help mitigate reproductive disorders and thereby improving fertility in animals as well as humans.

## Introduction

Pregnancy is a highly coordinated process in which higher energy demand is required for optimal fetal growth and development that may lead to overwhelming oxidative stress (OS) (Gupta et al., 2005). Evidence reveals that increased maternal oxidative stress contributes in numerous pregnancy disorders, such as preeclampsia, incomplete gestation period, reduced birth weight etc (Raijmakers et al., 2004; Scholl et al., 2005) and these anomalies known to relate with adverse outcomes rather than the consequences (Stein et al., 2008). Adequate supplementations of antioxidants are necessary to avoid OS; hence it works better than employing in adverse effects occurring during pregnancy. It indicates that ample amount of antioxidants are pivotal for maintaining a better level of antioxidants capacity.

Enhanced OS has been attributed to increasing metabolic rate, energy level and tissues oxygen requirements in normal human pregnancy (Idonije et al., 2011; Berchieri-Ronchi et al., 2015), sows, dogs and sheep (Vannucchi et al., 2007; Kim et al., 2013; Mutinati et al., 2013; Berchieri-Ronchi et al., 2015) respectively. Oxidative damages during pregnancy may predispose sheep to embryonic resorption, disruption in placental function, reduce fetal growth and induce stillbirths (Mutinati et al., 2013). The nutritional profile of gestation will influence pregnancy status imbalance their ratio, which ultimately causes fetal loss and other developmental outcomes (Villar et al., 2003).

Essential amino acids are known to be the key regulators of metabolic pathways as well as pivotal substances performing various functions such as growth, development, lactation and reproduction (Kim and Wu, 2004; Wu et al., 2010). A detailed review of the literature regarding the importance of dietary amino acids are needed to promote female fertility through amelioration of early pregnancy losses, whereas, animal model experiments on pigs and sheep are important for understanding the pregnant uterine environment (Bazer et al., 2012a; Bazer et al., 2012b).

Meanwhile, some amino acids produced in the body and their production is imbalanced under various stress conditions. These amino acids mediate metabolic pathways for development, lactation and reproduction. Functional amino acids are to be used to alleviate and control intrauterine growth restriction (IUGR) and infertility problems (Wu, 2013). The positive effect of amino acids and protein supplementation and their impact on fetal growth is well highlighted by (Herring et al., 2018). He also mentioned the inadequate or excessive intake of amino acids or protein in animal and human models. The present manuscript focuses on the importance of dietary amino acids supplementation and nutrient signaling pathways during pregnancy in females.

### Placental Oxidative Stress During Pregnancy

The placenta is responsible for performing several functions including nutrient exchange among mother and fetus (Wu et al., 2006). The growth of placenta is pivotal for optimal fetal development (Bazer et al., 2012). The placenta exerts two main functions in all species. First, it creates enormous space for the exchange of nutrients through forming the chorioallantoic placenta. Second, trophoblast cells within uterus to secrete histotroph which is crucial to meet the fetal energy requirement (Cross et al., 2003). The uterine environment of ruminant is equipped with secretary uterine glands and caruncles. The crancules varies between species (Bazer et al., 2012; Furukawa et al., 2014). During pregnancy, maternal crancules and fetal cotyledons unites to form placentome, performing high throughput nutrient supply from uterus to fetus. Disruption in placental development leads to fetal loss due to unsupply of hematotrophic nutrition (Reynolds et al., 2005). Placentome vasculogenesis is developed in early gestation because the majority of the placental growth seems to appear in the first trimester of pregnancy (Reynolds et al., 2005). Enhanced vascular changes support placentome to adopt the maternal environment for the nourishment of fetus (Borowicz et al., 2007). All these processes display a key role in enhancing blood flow percentage during the entire gestation (Vonnahme et al., 2007).

The transfer of amino acids through the placenta is pivotal for fetal growth. The reduction in amino acids transfer results in fetal growth restriction that might have adverse effects on fetal life. The amino acids passes through the placenta in order to cater the cumulative needs exchange and facilitate transporters to coordinate their function. It has been postulated that transporters alone could not be able to identify amino acid transfer while the factors which influence substrate availability blood flow and metabolism can limit the rate of transferring activity. To figure out rate-limiting property, it is essential to adopt advance tools which coordinate each other’s function. Emerging technologies enable the capacity to deliver compounds through targeted approaches within placenta to support slowly developing fetuses. Several factors are important for transferring amino acids and new tools are required to address hit rate-limiting factors (Cleal et al., 2011). More witnessed evidence of amino acids transfer during pregnancy has been descriptively reviewed by (Vaughan et al., 2017; Wu., 2010; Cleal et al., 2011).

Progression of normal placental development enhances the overloading of free radicals which are responsible for inducing OS, where placental mitochondrial activity causes free radical development (Myatt and Cui, 2004). Low level of antioxidants and oxygen environment requires for optimum embryonic development, and thus embryos make susceptible to oxidant products (Dennery, 2010). ROS at the physiological level can accelerate the growth of cells and effectively act as a signaling molecule to regulate embryonic development. Low level of oxygen environment during early pregnancy is responsible for triggering cellular functions for regulating angiogenic factors (Tuuli et al., 2011). The primary endogenous enzymatic system balances homeostasis and keeps ROS within the limit (Coughlan et al., 2004). It has reported that NAD(P)H oxidase may function as an oxygen sensor, responsible for differentiating cells despite excessive oxygen as well as vascular endothelial growth factor A (VEGF-A) and metalloproteins which are susceptible to oxidative damages (Poston and Raijmakers, 2004). Once, the fetoplacental unit is well established at the end of the first phase of pregnancy, a higher supply of oxygen due to the exceeding level of ROS by syncytiotrophoblastic cells were noted (Burton et al., 2010; Burton and Jauniaux, 2011). The metabolic activity in the body gradually increased, which in turn increases ROS levels that make enable to the risk of promoting OS. OS leads to the conversion of protein carbonyl to nitro, which can change the function of proteins, involving a series of factors such as placental enzymes, receptors, transporters, signaling molecules, structural proteins and hormones (Webster et al., 2008). OS is likely to be a cause of some pregnancy-related illnesses, such as spontaneous abortion, fetal growth restriction and low birth weight (Poston and Raijmakers, 2004). It has been confirmed in humans that progressive OS induces female reproductive diseases, including gestational diabetes, polycystic ovary syndrome, endometriosis, and preeclampsia (Duhig et al., 2016; Lu et al., 2018). The effects of progressive OS on reproductive performance of sows mainly reflected in reduced litter size and survival of piglets, decreased ability for lactation, and lower health status of sows (Prater et al., 2008). In addition to excessive ROS production in pregnant sows, decreased antioxidant system activity is also a reason for ROS accumulation in sows (Tan et al., 2015). Maternal high-energy diet-induced obesity may increase placental oxidative stress by up-regulation of Nox2 (Hu et al., 2019). Environmental stimuli such as high temperature and hypoxia can also cause free radical accumulation and damage the antioxidant system in sows (Zhoa et al., 2013).

Insufficient supply of nutrients during pregnancy in ruminants often exists due to excessive consumption of forages which make them vulnerable to alteration in nutrient availability and quality. There are several factors to be involved in transferring nutrients to the fetus such as placental growth, utero-placental blood flow etc (Dunlap et al., 2015). Providing lesser amounts of nutrition in pregnancy results in smaller offspring in comparison with adequately fed mothers (Satterfield et al., 2010). Under-develop fetal growth is the main obstacle to livestock producers, particularly due to nutritional scarcity in the time of drought. The adaptability of the placenta in such period faces environmental challenges impair nutrient transport for the growing fetus. To get know such gaps, there is a need for improving our understanding of placental nutrient transport which is crucial for management strategies to overcome fetal losses. Higher number of fetuses may affiliate to placental problems which negatively influence birth weight (Gootwine, 2005). Prolificacy trait is related with sheep production, but their economic impact has not been explored due to pregnancy and post-natal problems. Further, more factors which are believed to reduce birth weight such as maternal age, the season of the year, maternal body score and high altitude (McCrabb et al., 1992; Gootwine et al., 2007). Heat stress is known to cause declined placental birth weight (Tao and Dahl, 2013) and hence it influencing hormonal profile of placenta, where placental lactogen and pregnancy-associated glycoproteins are reduced (Thompson et al., 2013).

### Pregnancy Associated Problems due to Metabolic Disorders

Maternal obesity is globally accepted as a health concern issue. Data suggests that it affects all age groups, ethnicities and genders (Ogden et al., 2007). The possible factors to be involved are lifestyle, epigenetic factors consisting of food intake and physical activity (Mitchell et al., 2011). The link has drawn among obstetric complications in offspring, including the increasing evidence of perinatal morbidity. The nutrition environment of the uterus displays an essential role in fetal formation. Literature witnessed that over-nutrition of obese dam make offspring vulnerable to metabolic disorders (Dong et al., 2013). Fetal peri- and epicardial fat accumulation may raise myocardial energy utilization, where glucose used as an essential energy source. Moreover, aggregation of lipids also activates a robust energy pathway which further promotes other molecules (Stanley et al., 2012). Lipid possesses detrimental as well as beneficial effects on cardiac function. The latter one is to reduce inflammation and promote mitochondrial function (Stanley et al., 2012).

Several diseases can arise from suboptimal conditions of pregnancy (George et al., 2012). Fetal complications have been linked to metabolic problems which seem to appear in adult life (Risbridger et al., 2005). Restriction of nutrients during pregnancy can increase the response of glucocorticoid receptors in the newborn (Whorwood et al., 2001) that may alter the cardiac function later in life (Ponzio et al., 2012). The nutritive scenario during pregnancy regulates fetal heart function (Dong et al., 2008) and controls some gene functions for stimulation of the cardiac activity (Wang et al., 2010). In cardiac hypertrophy, insufficient regulation of glucose transporter, cardiac protein, and lipid accumulation may occur (Algahim et al., 2012). Nutritional status during the perinatal period may alter the protein levels crucial to glucose and lipid metabolism. The alternations in myocardial lipid metabolism protrude due to the nutritional status of the dam and influence cardiac associated nutrient sensor (Medina-Gomez et al., 2007). As a result, alteration in myocardial fatty acids, obesity and metabolic illness may rise in fetus later in life (Sullivan et al., 2011). The over-nutrition triggers fetal hormones, inflammatory cytokines which known to exert important function (Sullivan et al., 2011) although, the exact mechanism of cardiovascular dysfunction remains elusive.

Oxidative stress exerts an essential role in lipotoxicity associated changes in cardiovascular function (Ren and Kelly, 2009). The oxidative stress can develop by intracellular aggregation of triglycerides to suppress the mitochondrial efficiency and electron transport chain. Further, the proteomic profile of placenta exhibits the response of the altered protein against oxidative stress and inflammation in obese pregnant women. These problems are connected to fetal growth and development problems (Oliva et al., 2012). To identify the more impact of oxidative stress, Sen and Simmons offered antioxidant supplement to pregnant western diet-fed rats before offspring adiposity. The results indicate the enhanced adiposity with influenced glucose tolerance. The evidence of oxidative stress and inflammation were noted in preimplantation embryos, fetuses and newborns of western diet-fed rats. The gene response of pro-adiposity and lipogenic genes was modified in fat tissue of rats. Inclusion of antioxidants supplement reduced adiposity and brings glucose tolerance at the basal level (Sen and Simmons, 2010). It is worth noting the therapeutic properties of antioxidants in the regulation of postnatal adiposity with maternal fat, dietary exposure. Moreover, the antioxidant effects of dietary amino acids in this scenario may provide a beneficial effect.

Literature indicates that negatively regulated prenatal environmental often induces long term consequences of metabolic illness. Thus, it presumed that fetal programming also influenced by this mechanism due to the release of adipocytokines in conjunction with insulin resistance to trigger the inflammatory response (Vickers, 2007). It also increases the incidence of obesity in genetic polymorphism of adipokine genes and other molecules (Yu et al., 2012). Moreover, in obesity condition, higher plasma adipokines such as IL-6, CRP, TNF-α have been demonstrated (Ouchi et al., 2011). Growing evidence shows that obesity is accompanied by low-grade inflammation which impairs metabolic function. Adipose tissue serves as an essential endocrine organ through producing a variety of factors including pro- and anti-inflammatory cytokines (Ouchi et al., 2011). The obese pregnancy categorized by elevations in adipokines and inflammatory markers (Stewart et al., 2007). Increased physical mass in gestation (maternal obesity) denote higher chances of metabolic syndrome in offspring (Boney et al., 2005). The current scenario, indicate that leptin mediates postnatal fetal programming (Vickers, 2007) and providing new therapeutic options either from the maternal or neonatal site, which can target with nutritional manipulation in postnatal life. Further, more detailed description of metabolic illness of pregnancy is well described somewhere else by (Dong et al., 2013). Antioxidant activities of dietary amino acids in this condition need to be investigated to find out beneficial effects on pregnancy problems regulated by metabolic diseases.

### Contribution of Nutrient Sensing System in Reproduction

#### Mammalian Target of Rapamycin (mTOR) Pathway

**FIGURE 1:**
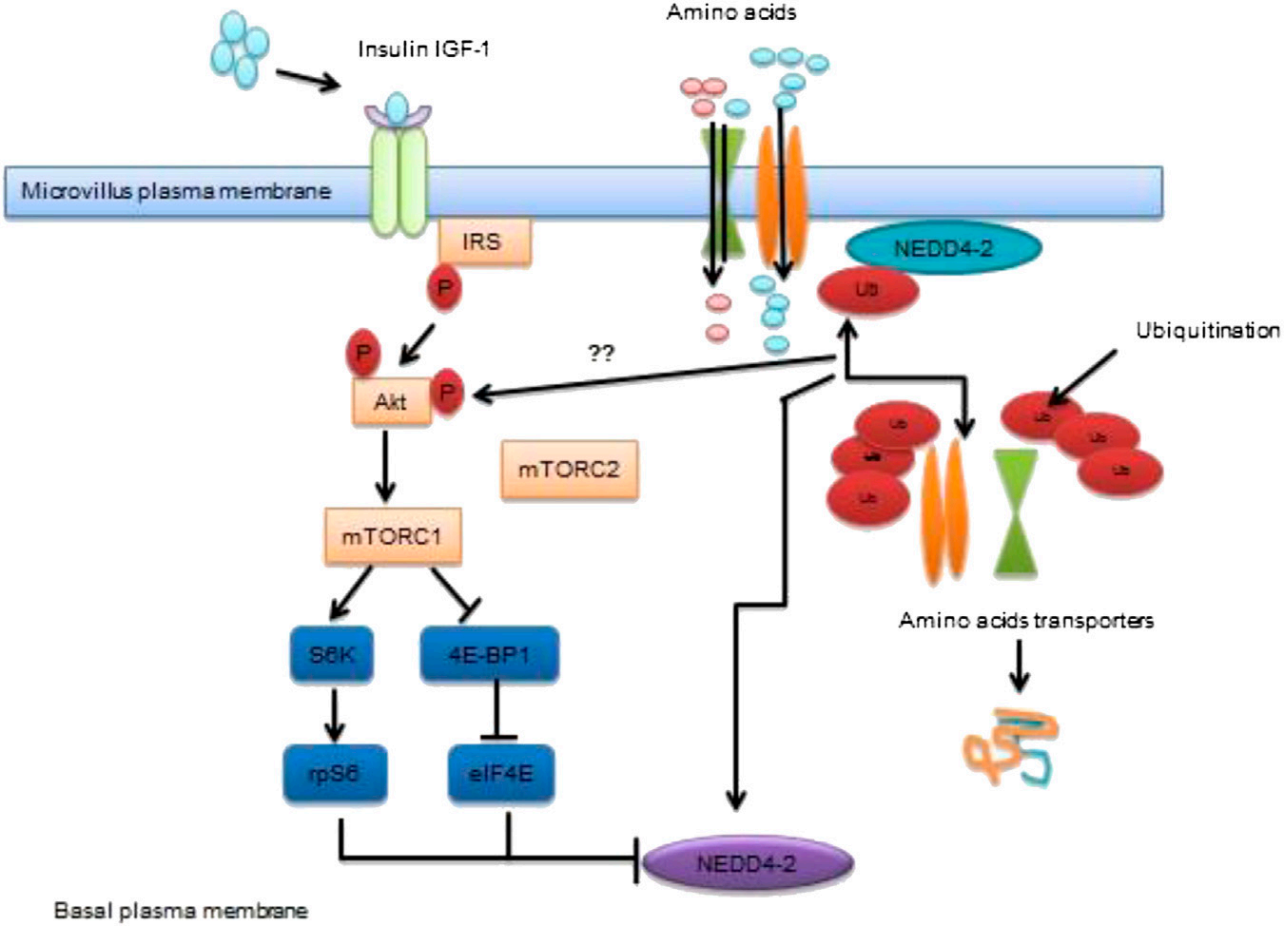
Transportation of amino acids to placenta via mTOR signaling pathway Schematic diagram of mTOR pathway of placenta act as a nutrient sensor. It co-ordinates nutrient and growth factors to modulate transfer of maternal nutrients to the fetal circulation. MTORC1 modulates trafficking of AA transports by employing differential ubiquitination mediated by NEDD4-2. Inhibition of NEDD4-2 leads to enhanced localization of AA transporters in the plasma membrane.

The mTOR pathway is a conserved region of the nutrient-sensing pathway serve as an important function in reproduction and longevity processes. The mTORC 1 (mechanistic target of rapamycin, complex 1) stimulation mediates glucose metabolism, lipid synthesis and mitochondrial function (Johnson et al., 2013; Saxton, and Sabatini, 2017). It combines the different high availability of nutrient and energy sensors to control metabolic functions important for directing energy homeostasis and reproductive status etc. The contribution of mTOR in age and reproductive weakening have not been largely investigated, failure mTOR function or upstream signals of mTORC-1 effector S6 K results in declined progeny production and or shifted reproduction process in C.elegans (Vellai et al., 2003; Pan et al., 2007). It also interrupts cell cycle of larval germ cells and decreased germline stem cell proliferation (Korta et al., 2012). Evidence indicates that the activation of mTORC-1 signals in granulosa cells mediates primordial follicular stimulation and potentiates the stimulation of phosphatidylinositol 3-kinase (PI3K) and Akt/Protein Kinase B pathway in oocytes (Zhang et al., 2014). The granulosa cells of the mouse are activated by mTORC-1 triggers ovulation and reproductive function (Huang et al., 2013). Overstimulation of oocyte-specific mTORC-1 induce primordial and growing follicles results in globular follicular activation and thereafter depletion follicles and other anomalies (Adhikari et al., 2009; Adhikari et al., 2010) drawing a relationship of female mice having influenced transcription factors (Hosaka et al., 2004; Reddy et al., 2008). Moreover, the stimulation of PI3K/Akt and mTORC1 signals along with their downstream targets may overlap within oocytes (Adhikari et al., 2010; Adhikari et al., 2013). The mTORC-2 also affects follicular activity and the source of oocyte-specific ablation of mTORC-2 component Rictor results in huge follicular apoptosis, inactivity of ovarian functional follicles, and imbalance in sex hormones and subsequently leads to premature infertility in mice (Chen et al., 2015). Furthermore, these wider actions of mTOR signals seem to be centrally regulating reproductive function/status (Roa et al., 2014). The role of mTOR signals in controlling reproductive processes is well-depicted in Figure 1.

Published literature revealed that mTOR signaling behave as nutrient sensing molecule contributing to placental function and fetal growth during late pregnancy (Dimasuay et al., 2016). In addition to that, mTOR signals in women having obesity/ gestational diabetes mellitus (GDM), given birth to larger babies and in animal models related to overgrowth of the fetus (Perez-Perez et al., 2013; Capobianco et al., 2016). Evidence has shown that exposure of *in utero* GDM to female offspring can be used as a GDM model in which irregularities in placental signaling become apparent due to overwhelming of ROS mediated pro-inflammatory response resulting in fetal overgrowth (Capobianco et al., 2016). Moreover, fetal overgrowth is a common complication of GDM associated with changing in growth factors and constantly surge the level of placental nutrient transport (Hiden et al., 2009). The phosphorylation of mTORC-2 finds different targets consisting of serum and glucocorticoid-inducible 96 kinase 1 (SGK-1), which play an essential contribution in the implantation process (Lou et al., 2017). However, the involvement of mTOR signals has been reported in regulating implantation, trophoblast and embryonic growth (Gardner and Harvey, 2015) while the activity of mTOR signals during early post-implantation period needs to be investigated.

### Kelch-like Ech-Associated Protein 1 (Keap1-Nrf2-ARE) Pathway

The Nrf2/ARE is a signaling molecule display key role in cellular defense in response to oxidative damages (Nguyen et al., 2009; Kumar et al., 2014). Along with that, two more molecules are needed to exert protective response and cytoprotective enzymatic effects such as antioxidant response elements (AREs) and kelch ECH linked protein 1 (keap1), which attaches Nrf2 within cytoplasm and enhancing proteasomal degradation of Nrf2 (Itoh et al., 1999; Nguyen et al., 2009). Keap1/Nrf2/ARE pathway exerting a key role in cellular defense towards OS and xenobiotic damage (Magesh et al., 2012). The Keap1/Nrf2/ARE also exerts an important role in progressing inflammatory response (Kim et al., 2010). Previous evidence showed that supplementation of amino acids attenuates inflammation arises due to the overproduction of OS and suppressing inflammatory cytokines. These beneficial effects of amino acids become prominent due to the interaction with several signaling pathways such as NF-κB, MAPK, Nrf2 and mTOR pathways (Fang et al., 2018). The p62 regulates Keap1-Nrf2-ARE signaling through kinase phosphorylation and the uncoupling of the protein kinase (Park et al., 2015). Collectively, activation of p62 cellular contact with Nrf2-binding site of Keap1, which inhibits interaction among Keap1 and Nrf2 and thus stimulation of several genes to exert antioxidant proteins and anti-inflammatory enzymatic response (Komatsu et al., 2010; Lau et al., 2010). Of note, that p62 stimulates Nrf2 while, Nrf2 may upregulate the expression of p62 signals, hence it showing a positive feedback loop. Considering the aforementioned literature, it is indicated that amino acid Trp could increase the expression of several molecules, like Keap-Nrf2-ARE and mTOR pathways. It has been witnessed that in extra-hepatic tissues, Trp may activate phosphorylation of p62 followed by stimulation of mTORC1. After that phosphorylated p62 uncoupling the relationship between Nrf2 and Keap1, resulting in stimulation of Nrf2 from the cytoplasm to the nucleus for transcription of antioxidant proteins and thus, promoting antioxidant cellular defense. Further, evidence is needed to identify the exact relationship between dietary intervention strategies of Trp and other amino acids with Keap1/Nrf2/ARE pathways (Kang et al., 2018).

### Peroxisome Proliferator-Activated Receptors (PPARs)

PPARs are conserved proteins; its stimulation takes place through ligands and depends upon the nature of the lipids and is responsible for regulating gene expressions of various metabolic processes (Wahli and Michalik, 2012). Apart from that, it also exerts important function on embryo organogenesis and fetoplacental growth and metabolism (Jawerbaum and Capobianco, 2011; Kadam et al., 2015). The literature reveals three forms of PPAR isoforms consisting peroxisome proliferator-activated receptor-gamma (PPARγ) and peroxisome proliferator-activated receptor delta (PPARδ) which exhibits significant role in embryonic development (Barak et al., 2008; Wahli and Michalik, 2012; Barquissau et al., 2017). Some placental defects in PPARγ and PPARδ null mice results in the death of 27 embryos indicating these forms are essential, and their absence via other PPAR isoforms rescue adverse phenotype (Wang et al., 2007; Barak et al., 2008). Previous literature reveals that decreased embryonic PPARδ and decidual PPARγ in diabetic rats during early organ development related to compromised embryo morphogenesis (Higa et al., 2010; Higa et al., 2014). Moreover, alterations in the fatty acid metabolism consist of massive crosstalk between PPAR and mTOR in different cell types have been documented (Blanchard et al., 2012; Angela et al., 2016) and all these nutrient pathways share target with fatty acid-binding protein-4 (FABP4) (Wang et al., 2017). Moreover, human placenta represents three isoforms of PPARs; however, their physiological function needs to be addressed. It further indicates that PPARγ and PPARβ seem to be involved in the crucial function of placental development (Fournier et al., 2007). PPARγ has been implied to control trophoblast differentiation and maturation (Schaiff et al., 2000) and also depict a key role in the regulation of fat accumulation in trophoblasts and transferring fatty acids from placenta to the fetus (Schaiff et al., 2006). Previous studies document the proposed involvement of PPARs in IUGR development. For example, Diaz et al. (2012) revealed that PPAR γ mRNA expression had suppressed in small placentas for gestational age (SGA) fetuses and its associated placental weight. However, treatments of dietary amino acids on PPARs mediated reproductive disorders, needs further investigation. This dietary aspect of amino acid supplementation might give some new insights and may help to alleviate PPARs regulated reproduction anomalies in the near future.

### Insulin/Insulin-Like Growth Factor Signaling (IIS) Pathway

**FIGURE 2:**
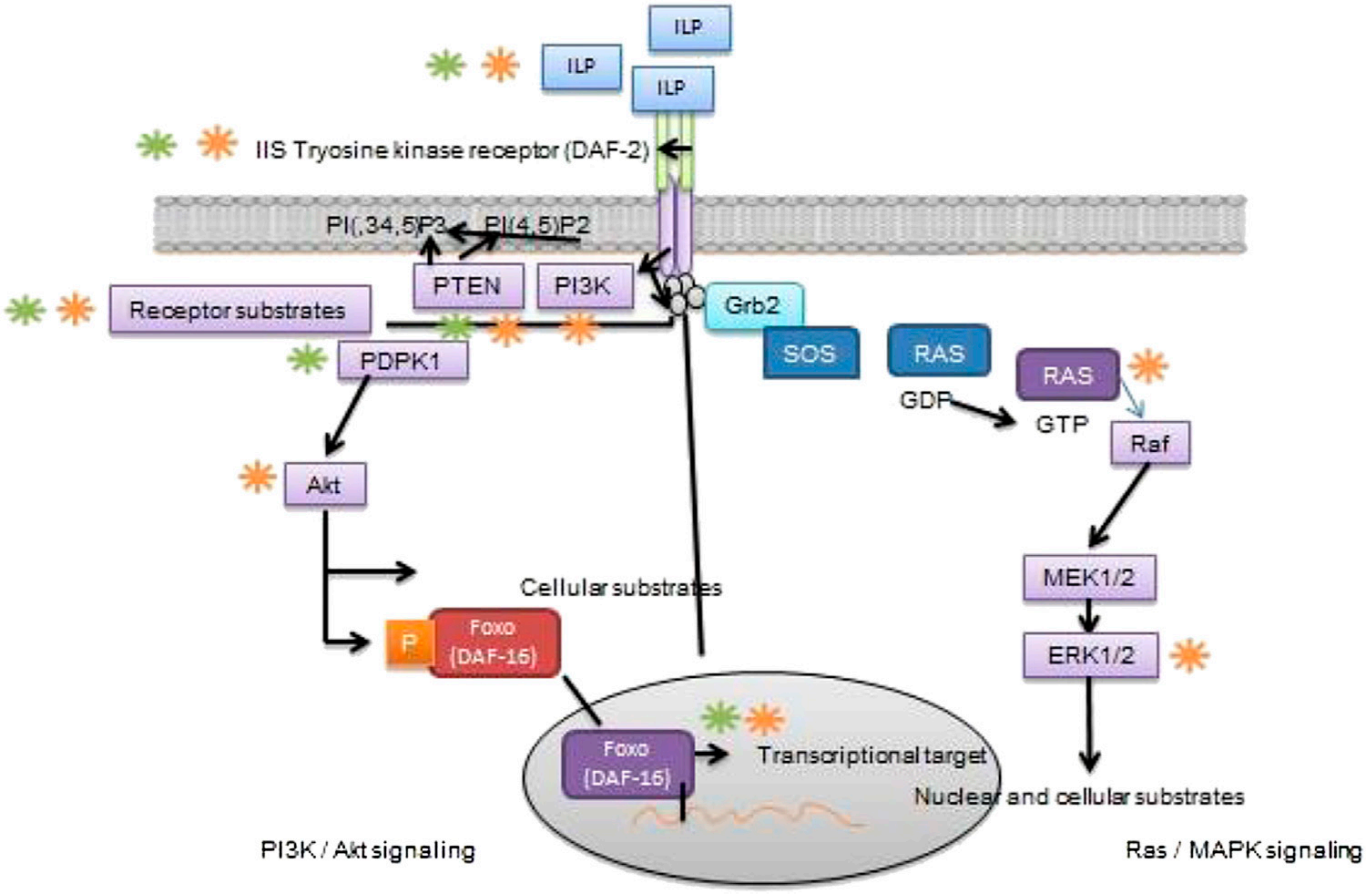
Role of IIS signaling pathway in reproduction and its longevity Schematic diagram elicits that IIS is actively involved in reproductive function and lifespan in C. elegans D. melanogaster or mice. In brief, ILP receptor binds with transmembrane for IIS tyrosine kinase receptor (DAF-2 in C. elegans), that autophosphorylates and recruits and finally stimulating key substrates, through different channels directly or via receptor substrate intermediate. Once the simulation of PI3k/Akt branch of IIS (left) takes place, it converts PI(4,5)P2 into second messenger PI(3,4,5)P3; which are negatively regulated by phospholipid phosphatases such as the PI3-phosphatase PTEN. Activation of PI (3,4,5) P3 causes recruitment 3-phosphoinositide–dependent protein kinase-1 (PDPK1) and its substrate, Akt/protein kinase B. Once the Akt pathway stimulates different cellular substrates not mentioned here, consisting of Rab-GTPase–activating protein AS160, glycogen synthase kinase-3, and TSC1/TSC2. The Akt is a key regulator of FoxO transcription factor (DAF-16 in C. elegans); that phosphorylates by Akt resulting in inhibition of transcriptional activity through bringing Akt back into the cytoplasm. On the other hand, Ras/MAPK branch of IIS (right), binds with adaptor protein Grb2 and the guanine nucleotide exchange protein Son of sevenless (SOS) to the activated IIS receptor, either directly or through docking protein, and hence it allows SOS to catalyze from inactive GDP-bound Ras to active GTP-bound Ras. The stimulation of small GTPase Ras activates of the serine/threonine kinase Raf, that leads to stepwise phosphorylation and activation of MEK1/MEK2 and then ERK1/ERK2. While the stimulation of ERK1 phosphorylates numerous cellular and nuclear substrates not display here, RSK and ELK1. Green color shows phosphorylation (kinase activity), whereas, (orange color) highlight dephosphorylating (phosphatase activity).

The IIS is a vital signaling molecule and a regulator of nutrient availability, energy homeostasis and metabolic processes in vertebrate and invertebrate species. The activation of IIS takes place through insulin-like peptide (ILP) ligands which are solely dependent on availability of nutrient and sensory information (Templeman and Murphy et al., 2017). The depletion of whole-body insulin substrate (IRS)-2 (Burks et al., 2000) defects in neuronal insulin receptors (Brüning et al., 2000) reduces fertility in mice due to the impairment in signaling network resulting in fewer or immature follicles. Of note, that oocyte-specific PDK1 signaling is needed for primordial follicle activity (Reddy et al., 2009). The impaired function of FoxO3A isoform or oocyte negative regulator PTEN (Castrillon et al., 2003; Reddy et al., 2008) causes stimulation of follicular pool resulting inactive ovarian follicles in female mice and thus develops infertility at early stage. Conversely, overexpression of FoxO3 enhances ovarian reproduction capacity and fertility in female mice (Pelosi et al., 2013). However, the different evidence report that the central and peripheral IIS display a pivotal role in the contribution of oocyte development, ovarian activity and reproduction status (Das and Arur, 2017; Sliwowska et al., 2014). It demonstrates that specific evolutionary conservative mechanism is involved which detects the oocyte quality maintenance (Hamatani et al., 2004; Luo et al., 2010) and we assumed, that it may impair reproduction and its ageing process in humans. Considering above witnessed findings, it concludes that IIS regulates reproductive longevity through involving transcription factors and processes which governs IIS function in reproduction and reproductive aging. The role of IIS in reproduction and its lifespan is shown in Figure 2. Although, the role of IIS in mammalian reproduction has not been well reported. The literature on this perspective is mainly focusing on invertebrate species but no studies have been conducted to identify the exact role IIS in mammalian reproduction and its associated problems. Furthermore, the dietary intervention of amino acids with IIS pathway needs to be addressed in fertility and its related issues.

### Adenosine Monophosphate-Activated Protein Kinase (AMPK) Signaling

**FIGURE 3:**
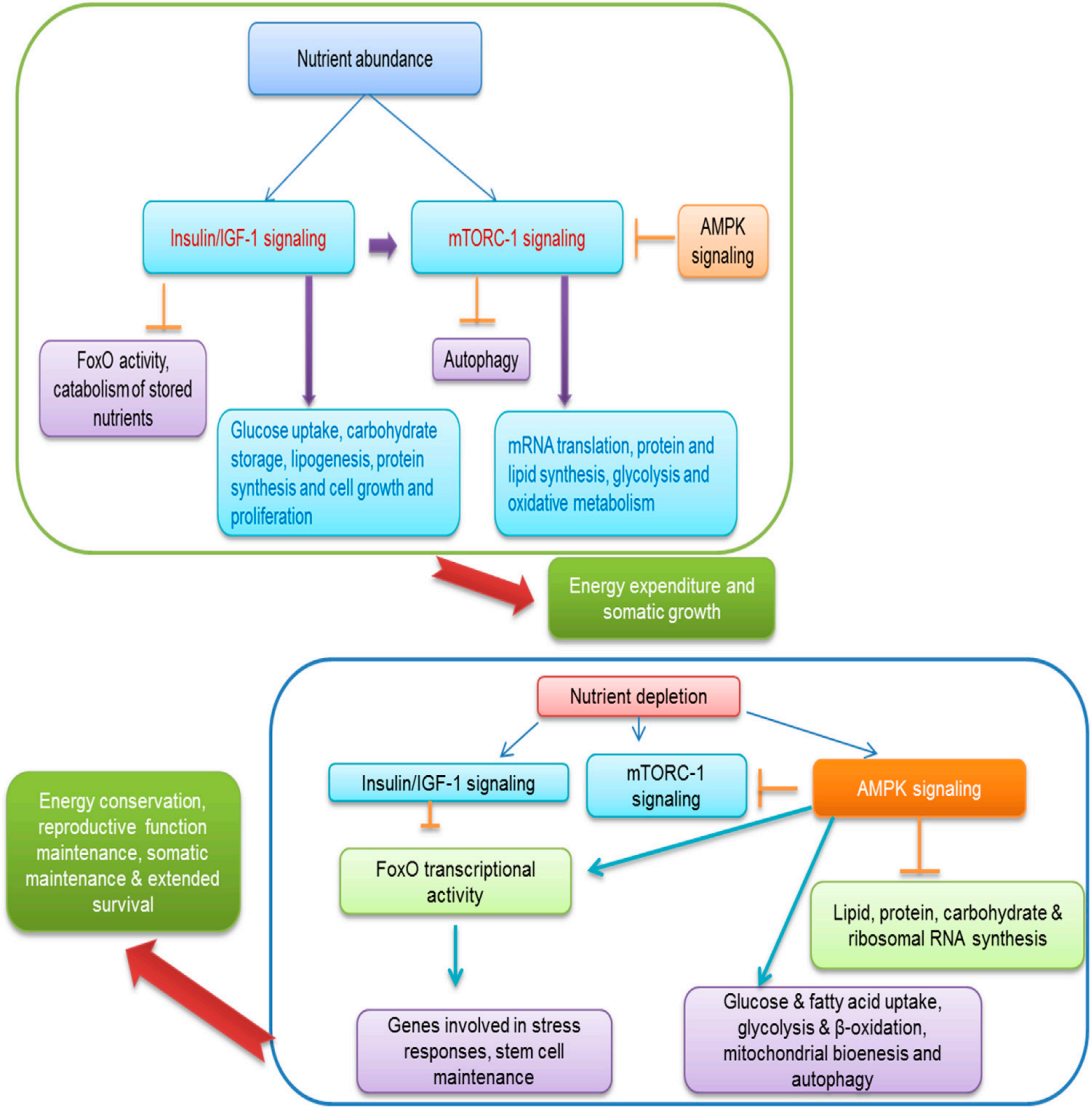
Beneficial effects of dietary supplementation of amino acids.

AMPK is a crucial nutrient-sensing molecule regulates energy and is activated in response to cellular energy depletion and induced catabolic and suppressed anabolic pathways respectively (Hardie et al., 2012; Hardie et al., 2016). AMPK consists of two regulatory subunits; kinetic function enhances either by direct AMP binding or by upstream kinases and responds once the levels of AMP, ADP, and/or calcium are elevated (Hardie et al., 2016). The AMPK enable to influence phosphorylating enzymes, regulatory proteins, and other cellular activities in a variety of metabolic processes (Hardie et al., 2012). For example, AMPK decreases protein synthesis and increases autophagy through suppression of mTORC1 (Hardie et al., 2012; Laplante and Sabatini, 2012). The indirect contribution of AMPK has been documented to alter protein expression levels in metabolic pathways via regulation of co-activators and transcription factors (Greer et al., 2007). In case of low energy level, excessive nutrient activates AMPK signals which subsequently increase energy production through regulating various energy contributors and also involved in downregulating signals via biosynthesis of different energy sources to suppress energy consumption (Hardie et al., 2012). In a more recent study, it depicts that Akt2-AMPK ablation highlighted high fat diet-induced cardiac irregularities via employing cGAS-STING-mechanism (Gong et al., 2020). Further, one more current finding evidenced that metformin possesses activity to trigger placental mitochondrial biogenesis and suppress epigenetic changes which seem to be presented in maternal diabetes during pregnancy showing health beneficial effects on offspring (Jiang et al., 2020). The previous investigation exhibits that AMPK deficiency promotes obesity triggered cardiac hypertrophy and impair contractile function perhaps due to the affiliation with AS160 and mTOR signaling (Turdi et al., 2011). The contribution of AMPK regulates reproduction, and its survival maintains energy homeostasis and metabolic pathways which perform a pivotal function in carrying out reproductive function in mammals. The reproductive function regulated by nutrient sensors due to availability of nutrition is depicted in Figure 3. The *in vitro* evidence of rate granulosa cells along with AMPK activating adenosine analogue alter the protein expression levels of the cell cycle (Kayampilly and Menon., 2009) and suppress progesterone secretion (Tosca et al., 2005) showing AMPK inhibits granulosa cell proliferation and sex hormone production. As discussed above that IIS and mTOR signaling, AMPK influence brain to centrally affect reproductive processes through mediating hormonal response and thus modulates hypothalamic-pituitary-gonadal axis and hampering estrus cycle and onset of puberty in animals (Roa and Tena-Sempere., 2014). The role of AMPK in animals is not largely known to influence reproductive function and its related problems. Interaction of dietary amino acids with AMPK needs to be elucidated further.

**FIGURE 4:**
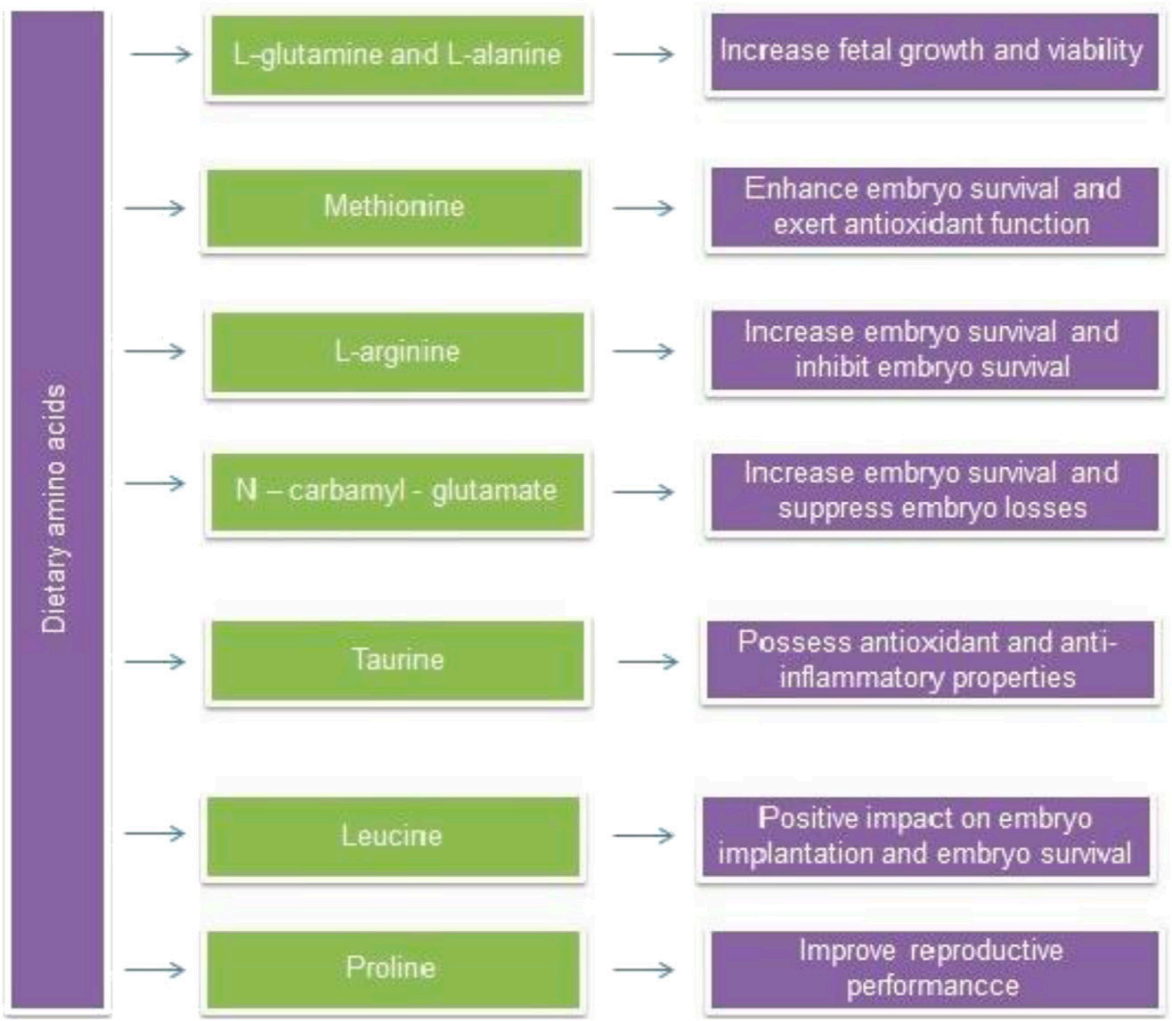
Regulation of energy homeostasis, reproductive function by nutrient sensing system in response to nutrient availability.

### Amino Acids Transportation to Fetus by Placenta

The fetal requirements of amino acids by placenta not only consisting of essential amino acids but also nutritionally non-essential amino acids (Wu et al., 2013; Wu et al., 2014). Glutamate-glutamine considered as a key player in late-pregnancy of pigs. *In vivo* evidence indicates that umbilical uptake of amino acids is a crucial source for supplying energy to the fetus. The placenta of sows show a higher concentration of glutamine during 20–40 days of gestation and increased concentration has been observed between 40and60 days of gestation and thereby declined simultaneously (Wu et al., 2013). Glutamate and alanine concentrations were constant in the porcine pregnant placenta (Self et al., 2004). Fetal accumulation of glutamate/glutamine reached a higher level on day 60–114 then followed by glycine, proline, hydroxyproline, aspararte/aspragine, leucine arginine, alanine and lysine respectively (Wu et al., 1999). The beneficial effect of some dietary amino acids on pregnancy is displayed in Figure 4.

The fetal plasma levels of amino acids do not alter during entire pregnancy while the positive impact has been documented than the maternal concentration (Philipps et al., 1987; Cetin et al., 1988). Thus, it shows a significant impact of amino acids transport from fetal to the maternal side. Moreover, in physiological pregnancies, a linear relationship of maternal to fetal ratio amino acids indicating that maternal flow of amino acids via umbilical cord has been increased (Cetin et al., 1988; Cetin et al., 1996). The syncytiotrophoblast is the central player for regulating transplacental amino acids passage and it is based on the influx of amino acids across the microvillous plasma membrane (MVM), the passage towards the cytoplasm of trophoblasts, from where it goes outside over the surface of trophoblast passes the basal membrane (BM) into the fetal circulation (Avagliano et al., 2012). Of note, placental amino acid transporters showed their presence over the surface of microvillus and on BM. The transportation mechanism of syncytiotrophoblast needs the energy to respond towards concentration gradient. On the other side, outflux of amino acid when passes the BM, Na+-independent systems display essential function. The transport system of amino acid passes the BM and it can be regulated via amino acid exchangers (takes one amino acid molecule from outside and one from inside and switches their position within the cell). Besides, availability and efficacy of few amino acids efflux transporters such as T-type amino acid transporter (TAT1), Na+-independent neutral l-amino acid transporter (LAT3) and System L amino acid transporters (LAT4) in the human BM were documented from isolated perfused human placental cotyledons (Cleal et al., 2011) such condition showing that diffusion may be possible by employing syncytiotrophoblast BM.

### Effect of Amino Acids Supplementation in Reproductive Disorders

Dietary amino acids can be viewed as nutritionally non-essential elements that are synthesized at sufficient concentrations to fulfill the body’s protein requirements. In general, dietary L-arginine is known to be indispensable for neonatal growth and survival (Wu et al., 2012). Other, amino acids, for example, glutamine, glutamate, and arginine play a pivotal role in modifying gene expression, cell signaling pathways, antioxidant response and help in boost up immunity (Yao et al., 2008; Brasse-Lagnel et al., 2009). In addition, glutamate, glutamine and aspartate are considered as key fuels in regulating metabolic functions. In contrast, essential amino acids including leucine, which stimulates mTOR to enhance protein formation, to avoid proteolysis while, tryptophan modulate immunological functions through activation of metabolites (e.g.kynurenine, serotonin and melatonin).

Autophagy is a natural process to replace damaged cells with the new one. By employing this process, damaged organelles, superfluous proteins and pathogens are confined and condensed into autophagosomes before merging with lysosomes for degrading and recycling. Autophagy is well recognized as protective and deleterious in malignancies. In amino acids insufficiency, the tumor cells rely on autophagy which attempted a direction to figure out inside story of autophagy regulated mechanisms where exposure of amino acids degrading enzymic based strategies into the therapeutic avenues of autophagy in cancer. A comprehensive review investigating the role of autophagy in cancer at cellular and molecular levels and significance of amino acids in regulating the autophagy process is excellently reviewed by (Ajoolabady et al., 2020). The author descriptively determines the role of autophagy in cancer, amino acids based abundance or derivation regulating autophagy using mTORC1 signals, enzymes or amino acids based strategies or kinase inhibitors on autophagy based cancer therapeutic options.

### Tryptophan

The Tryptophan (Trp) is considered as dietary essential amino acid and serves as a precursor of several molecules like serotonin, melatonin kynurenic acid etc (Liu et al., 2017). The literature revealed that Trp is well recognized exerting strong antioxidant properties via encountering free radicals, nitrogen reactive species, and chlorine species thereby, reduces cellular damage of free radicals (Reiter et al., 1999; Weiss et al., 2002). Moreover, animal evidence has been reported that dietary intervention of Trp supplementation could decline mortality, anti-abortive function in pregnant mice influenced by pseudorabies virus, and promoting fetal survival and viability (Xu et al., 2017).

### Glutamine

Porcine circovirus type 2 (PCV2) infections are categorized by loss of body weight, excessive growth of lymph nodes and diarrhea (Stevenson et al., 2001). Exposure of PCV2 infection during pregnancy may infect gestating pigs, resulting in abortion (Ladekjaer-Mikkelsen et al., 2001). Considering such hurdles, functional amino acids gained priority to improve immune status and disease resistance towards infections (Li et al., 2007; Ren et al., 2012) exerting strong antioxidant properties which can be a therapeutic intervention against pregnancy losses. Glutamine is conditionally essential amino acid depict prominent role in immune response, fetal growth, survival and metabolic regulation (Rogero et al., 2010). It exerts a number of functions including maintenance of cellular redox status and antioxidative functions etc (Xi et al., 2011). Hence, it evidenced that glutamine may provide protective effects on reproductive failure induced by PCV2 viral infection.

A study was conducted on pregnant mice that were fed a diet containing 1.0% L-glutamine or 1.22% L-alanine (isonitrogenous control) and infected with PCV2 virus (2000, TCID50) during gestation. The findings revealed that the inclusion of glutamine modulates the abortifacient effect PCV2 virus inoculation in mice. These findings showed that dietary supplementation of L-glutamine and L-alanine increased fetal growth and development as well as fetal survival rates by reducing the effects of PCV2 virus infection. Thus, amino acid supplementation could be helpful during the gestation period in ruminants as well as in humans (Ren et al., 2013). The beneficial and harmful effects of NO have been reported in physiological and pathological conditions such as virus and bacteria (Battegay, 1995). Despite that arginine also causes the activation of hormones released by the anterior pituitary gland and placental lactogen in humans and animals and thereby conception is regulated.

### Methionine

Methionine is a key nutrient that is essential for protein synthesis and S-adenosyl methionine (SAM) production (Wilson et al., 2004). In other species, supplementation with appropriate amino acids such as arginine, glutamine, methionine, leucine, glycine, have shown positive effects on embryo and fetal survival and growth by mediating main signaling pathways and metabolic pathways (Wang et al., 2012; DelCurto et al., 2013). Moreover, it has been reported that alterations in the function of methionine have been reported in bovine transcriptomic level during pre-implantation embryos *in vivo* (Penagaricano et al., 2013). Of note, that extracellular methionine is not necessarily needed for DNA methylation in cultured blastocyst (Bonilla et al., 2010). However, alterations in gene expressions appeared due to the changes in DNA methylation may result in either embryonic death or developmental deformities in preimplantation embryos (Roos et al., 2009). It has been demonstrated that methionine supplementation encountered ROS-induced insult by enhancing GSH activity (Del Vesco et al., 2014). Similarly, it has reported that the restriction of methionine diet supplementation in animals causes mitigation of oxidative damages. Thus, it indicates that the restriction of methionine mimics the mitochondrial-mediated ROS generation (Caro et al., 2009). The anti-oxidative effects of methionine have been widely documented in various species but their particular focus on reproductive disorders has not been clarified. Thus, there is a need for methionine restriction studies in the perspectives of pregnancy-related anomalies.

### Arginine

The published literature reveals that experimental induction of IUGR can possible with blocking NO synthesis (Diaz et al., 2005). Hence, NO donors (glyceryl trinitrate and isosorbide mononitrate), precursors (L-arginine) and NO mediator (sildenafil citrate and vardenafil) can play a possible role in therapeutic interventions against IUGR. Dietary supplementation of L-arginine or sildenafil citrate during pregnancy was reported to manage IUGR and its complications in clinical conditions (Chen et al., 2016). Dietary sources of L-arginine include seafood, tofu, spinach, sesame seeds, turkey, pork, beef, and dairy products. Dietary amino acids and their protective effects are shown in Table 1. In one study, sprague-dawley rats received dietary supplementation with arginine at 1.3% L-arginine-HCl or 2.2% L-alanine (isonitrogenous control) as a control for 1–7 days of gestation. As a result, arginine increased embryo survival in terms of litter size up to 30% at birth. Therefore, arginine supplementation during pregnancy could be helpful in avoiding early embryonic losses and thereby, enhancing reproductive performance (Zeng et al., 2008).

**TABLE 1:** Dietary supplementation with amino acids and pregnancy outcomes.

Amino acids	Model infective agent	Effects	References
1.0% L-glutamine or 1.22% L-alanine	10th day of PCV2 gestation KM mice	↑ survival and growth of embryos, fetuses, and neonates	Wu et al. (2013)
0.6% arginine	7th day of PCV2 gestation KM Mice	↑ litter number, litter birth weight and daily weight gain in neonates and partially inhibited reproductive insults	Ren et al. (2012)
1% L-arginine	30th days of -sows	↑ litter performance and immune response, and fetal growth in late gestation	Che et al. (2013)
Leucine	Fetuses of pregnant Walker 256 rats with or without tumor cells walker 256 tumor cells were assigned into six groups	Tumour growth reduced fetal weight. Oxidative stress was observed in W fetuses. Leucine supp. can be used for protection of fetal muscles	Cruz and Gomes-Marcondes (2014)
3.0% L-phenylalanine	days11–20 of the PKU induced experiment in a rat by DL-model of maternal α-methyl PKU phenylalanine (AMPhe)	↑ fetal amino acid profile and inhibited the impairment of fetal brain growth	Austic et al. (1999)
L-arginine & antioxidant vitamins	Humans -	reduced preeclampsia incidences in high risk population	Vadillo-Ortega et al. (2012)
Mixture of 18 amino acids	Humans (*in vitro* culture) -	improve viable and single embryos	Kim et al. (2007)

KM, Kunming mice; PCV2, Porcine circovirus type 2; PKU, Phenylketonuria.

### N-Carbamyl Glutamate (NCG)

A study was conducted on Ishikawa and JAR cells to explore the mechanism of N-carbamyl glutamate (NCG) on embryonic implantation (Zeng et al., 2012). Dietary supplementation with NCG enhanced serum levels of arginine, ornithine, glutamine, glutamate, and proline. Furthermore, activation of leucocyte inhibiting factor (LIF) expression promoted the signaling transduction of transcription 3 (Stat3), protein kinase B (PKB), and ribosomal protein S6 kinase (S6K1) during the peri-implantation period, leading to increase in litter size but not birth weight. Moreover, the addition of arginine and its metabolites to trophoblastic JAR cells promoted the expression of Stat3, PKB, and S6K1 which enhance cellular adhesion activity. Dietary NCG exposure during pregnancy can improve the reproductive efficiency of the animals. A study by Jinlong et al. (2015) demonstrated that NCG supplementation alters the endometrial proteome patterns resulting in improvement of embryonic survival and development in gilts. Moreover, other evidence showed that the dietary approach of NCG and rumen-protected arginine (RP-Arg) in underfed ewes provides a positive impact on a fetus in early-to-late gestation (Hao et al., 2016). Another study demonstrated that NCG supplementation at 500 mg/kg during the whole pregnancy period significantly provide beneficial effects on pregnancy outcomes in gilts (Zhang et al., 2014).

### Taurine

The consumption of alcohol outcomes during pregnancy is known as a fetal alcohol syndrome which causes developmental delay and influence on body growth, head and face abnormalities. Meanwhile, animal evidence denotes that maternal alcohol exposure reduced fetal viability and affects the development of growing embryo (Ghimire et al., 2008) whereas; high consumption of alcohol reduced the risk of birth weight, preterm birth and small gestational age (Patra et al., 2011). A study was revealed by (Ananchaipatana-Auitragoon et al., 2015) to explore the protective effects of taurine against maternal alcohol consumption and its effects on offspring. Exposure of 10 % alcohol, with or without maternal taurine supplementation during gestation and lactation to ICR-out bred pregnant mice. Adult offspring have shown that taurine supplementation reverted learning and memory damages. The results demonstrated that offspring of maternal alcohol exposure, together with maternal taurine supplementation reported conserved learning and memory while offspring with taurine supplementation indicates negative effects later in life. Taken results together, it highlights that taurine exhibited neuroprotective effects and preserves learning and memory processes once they were given with alcohol consumption.

Taurine is widely available free amino acid in mammals and displays key role in antioxidant and anti-inflammatory activities. Two sources are responsible for the synthesis of taurine such as absorption through diet and by cysteine metabolism (Stipanuk, 2004). Taurine protective effects were noticed in animal models against oxidative damages (Sinha et al., 2007). Whereas; antioxidation effects have been linked with ROS scavenging effects. Mounting evidence reported that taurine inhibits over-stimulation of mitochondrial Ca2+ absorption. Moreover, taurine also enhances antioxidant enzymes in oxidant-induced models. Thus, the study confirms that taurine ensures protection against oxidants through enhancing Mn-SOD and GSH-Px in mice mitochondrion after tamoxifen infection (Parvez et al., 2008). Various studies proved significant antioxidation effects in numerous models but their effects on the disruption of oxidant triggered pregnancy needs to be evaluated.

### Leucine

Leucine is a dietary amino acid which body cannot synthesize it (Giessen et al., 2007) and supplied through the diet. Its metabolism mainly related to early embryonic development through the activation of the mTOR pathway (Teodoro et al., 2012). It exerts a key role in blastocyst development that may further proceed to embryonic implantation (Zhang et al., 2017). In a study by Liu et al. (Liu et al., 2018) was revealed that premating dietary supplementation of 0.6% leucine in SD rates for two weeks have increased reproductive performance, maternal antioxidative capacity, and immune status in primiparous rats. Thus, improvement in reproductive performance and immune function may be attributed with amelioration of oxidative damage via leucine supplementation.

### Proline

Non-essential amino acids such as arginine, proline and glutamine in conjunction with few essential amino acids like leucine, tryptophan makes the group of functional amino acids that are known to regulate metabolic pathways to promote health, survival, growth and development, lactation and various reproductive processes (Wu, 2009). Disruption in the amino acid mechanism will lead to influence protein synthesis as well as body homeostasis (Wu et al., 2010). Functional amino acids in pregnant animals indicate an important role in the development of the placenta, conceptus linked with gestational structures (Wu et al., 2008) consisting of distinction of embryonic stem cells (Pistollato et al., 2010). These groups of amino acids regulate protein synthesis, polyamines and NO during entire pregnancy (Andrade et al., 2010). The synthetic rate of polyamines and NO greatly increased in early pregnancy (Teodoro et al., 2012) thus it is not only pivotal for embryogenesis but also a key player in optimum placental growth, angiogenesis and blood flow and therefore, it transfers to nutrition and oxygen to the developing conceptus (Wu et al., 2006). Disruption in the function of placenta thus affects its ability to supply nutrients, oxygen to the fetus and leading to embryonic death and IUGR. Studies conducted by Gonzalez-Añover et al. (2017) has shown that short term supplementation of L-proline around implantation stages increased reproductive efficiency and prolificacy of first and second parity sows. The results highlight that L-proline supplementation enhances litter size and birth weight which is potentially connected with maternal characteristics, for example, parity, prolificacy and the cost-effective supplementation having high energy balance.

Inadequate feed intake is a major factor for lactating sows before breeding due to utilization of internal reserves of nutrients for milk production leads to severe catabolic state and extends service period (Kraeling and Webel, 2015). Moreover higher intake of energy enhancing the ovulation rate in farm animals such as pigs (Kraeling and Webel, 2015).. Therefore, the increase feeding intake for short time before breeding (flushing), was implicated in previous reports to attempt an increase the numbers of embryo/fetuses (sheep) (Johnston et al., 2008). This practice does not work in swine breeding due to risk of embryonic death (Freking et al., 2016). Another investigation was reported that, higher intake of energy just before pregnancy/ in early gestation prone to embryonic mortality and declines embryonic growth in swine (Kraeling and Webel, 2015). The higher intake of proteins increased the levels of ammonia in plasma; and how ammonia affects embryonic survival and growth? It needs further investigation. Conversely, lower protein diet could not be able to fulfill the requirement for developing fetus and its outcomes due to inadequate supplementations of amino acids.

## Conclusion

Dietary amino acids have attracted tremendous attention due to their versatile role in female reproduction in terms of placental protein synthesis, angiogenesis, vasculogenesis and transfer of nutrients from the mother to the fetus via placental transporters. Nutritionally mediated placental growth and functions are considered to be effective for improving the embryonic and fetal prognosis. Furthermore, amino acids such as arginine, leucine, glutamine, and proline stimulate the mTOR pathway to increase protein synthesis and cell proliferation in the placenta to supply optimum nutrients to the growing fetus. Moreover, other signaling pathways which display important role in protecting pregnancy such as PPARs, IIS and AMPK, Keap1-Nrf2-ARE and mTORC1/Keap1-Nrf2-ARE. A little information on dietary amino acids intervention with these signaling pathways are available, but it needs to be further investigated to dig out underlying molecular mechanism which might be helpful for pregnancy development and its protection against oxidative damages. Dietary supplementation with L-glutamine or L-glutamine enhances nutrient conversion and enhances litter birth weight. Recent findings in ovine and porcine models indicate that there is an association between amino acids and polyamine pathway, which play a beneficial role in fetal survival during the intrauterine period. In addition, the metabolic pathways of dietary amino acids may provide insights regarding the development and survival of the fetus. The evidences reviewed here indicate that OS is actively involved in reproductive disorders and adverse developmental outcomes. Limited research has been conducted on antioxidative properties of dietary amino acids in reproductive disorders. Therefore, more insights and their molecular mechanism need to be explored. Dietary supplementation with amino acids can help prevent and control of reproductive disorders, which are prominent at adult stage in humans and mammals. Moreover, further research should be conducted on the effects of dietary supplementation of amino acids on pregnancy using different models to obtain insights that might contribute to successful pregnancies.

## Author Contributions

All authors have made a substantial, direct, and intellectual contribution to the work and approved it for publication.

## Funding

This project was funded by the the National Natural Science Foundation of China (U20A2054) and Hunan Innovation and Entrepreneurship Technology Investment Project (2019GK5016). We are also thankful to CAS-TWAS President's Fellowship and financial and infrastructure support from UCAS.

## Conflict of Interest

The authors declare that the research was conducted in the absence of any commercial or financial relationships that could be construed as a potential conflict of interest.
